# Prophylaxie post-exposition contre le virus Ebola : arguments en faveur d’un élargissement du rôle des anticorps monoclonaux dans un contexte d’accès limité

**DOI:** 10.48327/mtsi.v6i1.2026.835

**Published:** 2026-03-31

**Authors:** Julien POTET, Ismael ADJAHO, Kerry DIERBERG, Armand SPRECHER, Rebecca Marie COULBORN

**Affiliations:** 1 Médecins sans frontières, Centre opérationnel de Paris, 14-34 avenue Jean Jaurès, Paris, France; 2 Médecins sans frontières, Centre opérationnel d’Afrique de l’Ouest et centrale, Lot 44 îlot 7, Marcory Biétry Zone 4c, Abidjan, Côte d’Ivoire; 3 Médecins sans frontières, Centre opérationnel de Bruxelles, Rue de l’Arbre Bénit 46, 1050 Bruxelles, Belgique; 4 Médecins sans frontières, Épicentre, Département d’épidémiologie d’intervention et formation, 14-34 avenue Jean Jaurès 75019 Paris, France

**Keywords:** Maladie à virus Ebola, Vaccin, Anticorps monoclonaux, Anticorps thérapeutiques, Immunothérapie passive, Prophylaxie post-exposition, Afrique de l’Ouest, République démocratique du Congo, Afrique subsaharienne, Ebola virus disease, Vaccine, Monoclonal antibody, Therapeutic antibodies, Passive immunotherapy, Post-exposure prophylaxis, West Africa, Democratic Republic of Congo, Sub-Saharan Africa

## Abstract

**Justification et méthodes:**

Alors que la maladie à virus Ebola (MVE) reste une menace majeure, des progrès récents ont permis l’homologation de plusieurs produits médicaux, en particulier le vaccin rVSVΔG-ZEBOV-GP (Ervebo) et deux traitements par anticorps monoclonaux, mAb114 (Ebanga) et REGN-EB3 (Inmazeb). Leur utilisation en prophylaxie post-exposition (PPE) reste cependant peu documentée. Notre revue narrative de la littérature et des lignes directrices proposées par des organisations en réponse aux épidémies d’Ebola vise à évaluer le potentiel du vaccin rVSVΔG-ZEBOV-GP et des anticorps monoclonaux mAb114 et REGN-EB3 pour une PPE suite à une exposition à haut risque d’infection.

**Résultats:**

Très peu d’études ont été menées spécifiquement pour déterminer l’efficacité des vaccins ou des anticorps monoclonaux en PPE. Les données chez l’animal et chez l’homme suggèrent que le vaccin rVSVΔG-ZEBOV-GP n’offre qu’une protection limitée en PPE, bien qu’il puisse réduire la sévérité de la MVE si elle survient. Aucune des 34 personnes ayant reçu le vaccin dans le cadre de la PPE à ce jour n’a développé de MVE. Une étude rétrospective cas-témoins suggère une efficacité de seulement 16 % contre la survenue de la MVE lorsque le vaccin est administré pendant une période compatible avec la PPE. En revanche, les anticorps monoclonaux mAb114 et REGN-EB3, en raison de leur action immédiate par immunisation passive, semblent conférer une protection plus élevée contre la survenue de la MVE après une exposition à haut risque, avec une survie à 100 % en modèle animal, bien que les données cliniques humaines restent rares avec seulement 23 cas d’utilisation recensés en PPE et aucune survenue de MVE.

**Discussion et conclusion:**

L’accès aux anticorps monoclonaux pour les membres de la communauté ayant fait l’objet d’une exposition à haut risque devrait être assuré, tout en optimisant les critères d’évaluation du risque. Des recherches supplémentaires, notamment sur l’interaction entre vaccination et administration d’anticorps, sont essentielles pour déterminer les stratégies de PPE les plus efficaces. Enfin, un stock d’urgence d’anticorps monoclonaux et une amélioration du suivi des contacts sont indispensables pour renforcer la réponse aux épidémies. Malgré les limites des données actuelles, les anticorps monoclonaux devraient être envisagés comme option prioritaire en PPE pour les personnes à haut risque.

## Abstract (en)

**Introduction and methods:**

While Ebola virus disease (EVD) remains a major threat, recent advances have led to the approval of several medical products, in particular the rVSVΔG-ZEBOV-GP vaccine (Ervebo) and two monoclonal antibody treatments, mAb114 (Ebanga) and REGN-EB3 (Inmazeb). However, their use in post-exposure prophylaxis (PEP) remains poorly documented. Our narrative review of the literature and guidelines aims to assess the potential of the rVSVΔG-ZEBOV-GP vaccine and monoclonal antibodies mAb114 and REGN-EB3 for PEP following high-risk exposure.

**Results:**

Very few studies have been conducted specifically to determine the efficacy of vaccines or monoclonal antibodies as PEP. Data from animals and humans suggest that the rVSVΔG-ZEBOV-GP vaccine offers only limited protection as PEP, although it may reduce the severity of EVD if it occurs. None of the 34 individuals who have received the vaccine as PEP to date have developed EVD. A retrospective case-control study suggests an efficacy of only 16% against the occurrence of EVD when the vaccine is administered during a period compatible with PEP. In contrast, the monoclonal antibodies mAb114 and REGN-EB3, due to their immediate action through passive immunisation, appear to confer higher protection against the onset of EVD after high-risk exposure, with 100% survival in animal models, although human clinical data remain scarce with only 23 cases of use reported in PEP and no occurrence of EVD.

**Discussion and conclusion:**

Access to monoclonal antibodies for community members following high-risk exposure should be ensured, while optimising risk assessment criteria. Further research, particularly on the interaction between vaccination and antibody administration, is essential to determine the most effective PEP strategies. Finally, an emergency stockpile of monoclonal antibodies and improved contact tracing are essential to strengthen the response to epidemics. Despite the limitations of current data, monoclonal antibodies should be considered a priority option for PEP after high-risk exposure.

**Figure:**
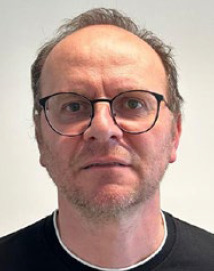

## Introduction et méthodes

La recherche de nouveaux outils pour lutter contre la maladie à virus Ebola (MVE) a fait des progrès considérables au cours des dix dernières années. Des essais cliniques portant sur plusieurs candidats vaccins et thérapeutiques ont été menés lors des épidémies à virus Ebola (EBOV) en Afrique de l’Ouest entre 2013 et 2016, et en République démocratique du Congo (RDC) entre 2018 et 2020. Aujourd’hui, il existe quatre produits recommandés par l’Organisation mondiale de la Santé (OMS) : deux vaccins et deux traitements par anticorps monoclonaux [21,22]. Parmi les deux vaccins homologués, celui qui a été priorisé en termes de réponse aux épidémies est le rVSVΔG-ZEBOV-GP, adapté à ce type de contexte en raison de sa formulation à dose unique capable d’induire une protection rapide [23].

En infectiologie, les anticorps thérapeutiques sont fréquemment utilisés à visée prophylactique, en association ou non aux vaccins [9]. Cependant, à l’heure actuelle, l’utilisation à but prophylactique des anticorps monoclonaux contre Ebola est très restreinte : des protocoles et recommandations existent, mais seulement pour les professionnels de santé victimes d’un accident d’exposition [20], et pour les nouveau-nés de moins de 7 jours nés de mère infectée par EBOV [22]. Leur utilisation parmi les membres de la communauté ayant fait l’objet d’une exposition à haut risque à EBOV n’est pas encore recommandée. Selon les définitions les plus récentes, en plus des nouveau-nés nés de mère infectée, il y a trois catégories d’individus considérés à haut risque : 1) les enfants allaités par une mère infectée par EBOV; 2) les individus en contact direct avec une personne atteinte d’une infection confirmée par EBOV présentant des symptômes dits « humides » tels que diarrhée, vomissements ou hémorragies externes, ou bien en contact avec ses fluides corporels; 3) les individus en contact direct avec le cadavre d’une personne atteinte d’une infection confirmée ou probable par EBOV [12].Dans cet article, en complément d’une revue de littérature récemment publiée [12], nous proposons d’analyser les données disponibles concernant le rVSVΔG-ZEBOV-GP et les anticorps monoclonaux mAb114 et REGN-3, dans la perspective de leur potentielle utilisation en prophylaxie post-exposition (PPE). Les caractéristiques principales de ces trois produits sont présentées dans le Tableau I. Notre revue narrative, basée sur des articles revus par des pairs et sur des lignes directrices proposées par des organisations intervenant en réponse aux épidémies d’Ebola, vise à répondre à la question suivante : quels outils seraient susceptibles d’être les plus efficaces pour une utilisation en PPE, le vaccin rVSVΔG-ZEBOV-GP, les anticorps monoclonaux mab114 et REGN-EB3, ou une combinaison des anticorps et du vaccin ? Nous détaillons ici les données disponibles et les questions encore en suspens, tout en reconnaissant que notre recherche bibliographique n’est pas nécessairement exhaustive et que d’autres médicaments, notamment de nouveaux antiviraux comme l’obeldesivir, sont eux aussi proposés comme des candidats pour la PPE. Les anticorps monoclonaux agissant comme une immunisation passive, nous soutenons qu’ils devraient être proposés après une exposition à haut risque à EBOV, non seulement aux professionnels de santé, mais aussi aux membres de la communauté.

**Tableau I:** Principales caractéristiques des produits envisagés pour la PPE de la MVE

	Disponibilité et coûts	Conditions de stockage	Durée de vie	Mode d’administration
**REGN-EB3**	Stock d’urgence aux États-Unis. Prix payé par le gouvernement américain estimé à au moins 6 900 US$ par traitement. Stock d’urgence de 500 traitements donné par Regeneron à l’OMS en septembre 2025	À conserver entre 2 °C et 8 °C	36 mois	Voie intraveineuse
**mAb114**	Pas de stock d’urgence à l’OMS Programme de donation de mAb114 développé par Ridgeback-Bio, mais origine des doses et quantités disponibles inconnues	À conserver entre 2 °C et 8 °C	Variable selon le lot utilisé	Voie intraveineuse
**rVSVΔG- ZEBOV-GP**	Stock d’urgence de 500 000 doses à l’OMS (ICG), financé par GAVI. Prix payé par GAVI : 99 US$ par dose	À conserver entre -80 °C et -60 °C, avec une tolérance de 14 jours entre 2 °C et 8 °C	36 mois	Voie intramusculaire

## Résultats

### rVSVΔG-ZEBOV-GP en PPE

Le vaccin rVSVΔG -ZEBOV-GP, commercialisé par Merck Sharp & Dohme (MSD) sous le nom de marque Ervebo, utilise le virus de la stomatite vésiculaire (VSV) comme vecteur réplicatif et porteur de l’antigène, la glycoprotéine d’EBOV (EBOV-GP). Il a été démontré que le vaccin, 10 jours après son administration, confère un niveau très élevé de protection contre la survenue de la MVE [10,16]. Le vaccin est essentiellement utilisé pendant les épidémies dans le cadre d’une vaccination dite « en anneau » autour d’un cas, parmi les contacts du cas et les contacts de ces contacts. De fait, dans les contacts recevant ainsi le vaccin, il y a des personnes récemment exposées à EBOV, dont un certain nombre a pu faire l’objet d’une exposition à haut risque. Il peut s’agir de membres de la famille du patient qui lui ont prodigué des soins sans aucun équipement de protection avant que le diagnostic ne soit posé, ou de participants à des rites funéraires non sécurisés pendant lesquels le corps du défunt a été touché.

Il y a des raisons de craindre que le rVSVΔG-ZEBOV-GP ait une efficacité limitée en PPE chez les contacts ayant été récemment exposés à EBOV, étant donné que les anticorps vaccinaux sont généralement détectés entre 7 et 14 jours après la vaccination chez la plupart des sujets [11], et que la durée médiane d’incubation de la maladie est estimée selon certains auteurs à 10 jours [14]. Cependant, il a été mis en évidence que le vecteur viral rVSV permet une stimulation de l’immunité innée qui pourrait rapidement conférer une certaine protection avant que la réponse immunitaire spécifique à l’antigène puisse faire son effet [17]. C’est ce que suggèrent les données animales : lorsque le vaccin est administré à des macaques moins de 24 heures après qu’une dose de 1 000 pfu d’EBOV, létale en l’absence d’intervention médicale, leur ait été inoculée, entre un tiers et deux tiers des singes survivent, selon les expériences [7,15]. La protection est non négligeable, mais elle est limitée.

Du côté clinique, 8 cas de professionnels de santé ayant reçu le vaccin rVSVΔG-ZEBOV-GP suite à des accidents d’exposition à EBOV à niveaux de risque variables ont été recensés en 2017. Aucune infection n’a été signalée [8]. Plus récemment, parmi 45 personnes ayant été en contact au Royaume-Uni avec une patiente précédemment guérie et souffrant d’une rechute de la MVE, 26 ont reçu le vaccin. Aucune infection n’a été détectée, ni dans le groupe recevant le vaccin, ni dans le groupe témoin. Les caractéristiques de la patiente index (un cas rare de rechute) étant très particulières, et le risque d’exposition étant considéré comme modéré, ces données doivent être interprétées avec beaucoup de prudence [6]. Une étude rétrospective cas-témoins à tests négatifs, menée en RDC entre 2018 et 2019 [16], permet d’y voir un peu plus clair. Cette étude se focalise sur des contacts ayant développé des symptômes évocateurs de la MVE et admis en conséquence dans un centre spécialisé de lutte contre Ebola pour une évaluation diagnostique et une prise en charge : le groupe « témoin » est constitué des contacts ayant finalement été testés négatifs à EBOV, alors que le groupe « cas » est constitué des contacts qui ont effectivement développé la MVE. Sans surprise, la proportion de personnes ayant reçu le vaccin plus de 10 jours avant la survenue des symptômes dans le groupe témoin (finalement négatif à EBOV) était très nettement supérieure à celle dans le groupe cas (finalement positif à EBOV). Les auteurs estiment ainsi que l’efficacité du rVSVΔG-ZEBOV-GP à prévenir la survenue de la MVE dans les 10 jours ou plus suivant la vaccination est de 84 % (95 % CI [70,92]). En revanche, la différence entre les groupes « témoin » et « cas » était bien plus faible chez les personnes vaccinées entre 3 et 9 jours avant la survenue de symptômes. La protection conférée par rVSVΔG-ZEBOV-GP contre la survenue de la MVE dans les 3-9 jours suivant la vaccination n’est ainsi estimée qu’à 16 % (95 % CI [35,48]). Si l’on considère que la durée moyenne d’incubation de la MVE est d’environ 10 jours [14], on peut considérer qu’un grand nombre de ces cas avaient été vaccinés après avoir été exposés à EBOV. Le faible échantillon et l’important intervalle de confiance appellent à la prudence, ainsi que la variation probable de l’efficacité du vaccin entre les personnes vaccinées 3 jours avant l’apparition des symptômes et celles vaccinées 9 jours avant. Néanmoins, ces données, considérées dans leur ensemble, suggèrent indirectement que le vaccin aurait une efficacité limitée contre le développement de la MVE s’il était utilisé dans le cadre de la PPE.

Cependant, une autre étude rétrospective en RDC, cette fois uniquement parmi des patients souffrant de la MVE, suggère que la vaccination récente, entre 3 et 9 jours avant l’apparition des symptômes, est associée à un risque de décès inférieur de plus de moitié au risque de décès observé chez les patients atteints de MVE non vaccinés (risque relatif ajusté : 0,44 [0,29–0,65, p=0,0001]) [4].

### REGN-EB3 et mAb114 en PPE

Différentes approches d’immunisation passive ont été proposées pour le traitement ou la prévention de la MVE, notamment à partir de sérums de patients convalescents ou de sérums d’origine animale [2,27]. Cependant, les deux traitements homologués contre la MVE sont constitués d’anticorps monoclonaux ciblant la glycoprotéine d’EBOV. Il s’agit du mAb114 (ansuvimab, nom de marque Ebanga, commercialisé par Ridgeback-Biotherapeutics) et du REGN-EB3 (atoltivimab + maftivimab + odesivimab, nom de marque Inmazeb, commercialisé par Regeneron), qui ont démontré tous deux leur efficacité en tant que traitement de la MVE. Ils doivent être administrés par voie intraveineuse.

Il a été mis en évidence, aussi bien chez l’homme que chez l’animal, que plus les traitements ciblant EBOV et les autres filovirus sont démarrés tardivement après le début des symptômes, moins leur efficacité est élevée [5,19]. Il existerait ainsi un gradient d’efficacité en fonction du temps. En suivant ce raisonnement, on peut faire l’hypothèse que l’efficacité du mAb114 et du REGN-EB3 à prévenir la MVE ou à en atténuer la sévérité si la maladie survient malgré tout, devrait être très élevée s’ils sont administrés en PPE.

Les résultats obtenus chez l’animal vont dans ce sens. L’administration de trois doses consécutives de mAb114 à des macaques rhésus 24 h, 48 h puis 72 h après qu’une dose de 1 000 pfu d’EBOV leur ait été inoculée, a occasionné un taux de survie de 100 %, sans la survenue de signes cliniques, mais sans empêcher toutefois la présence d’une très faible virémie [3]. Il s’agit ici à notre connaissance des seules données disponibles chez des primates non-humains avec un schéma expérimental mimant la PPE, selon lequel le traitement démarre très tôt après l’inoculation du virus et avant la survenue de symptômes. D’autres expérimentations avec le REGN-EB3 et le mAb114 ont été publiées chez des primates non-humains, avec un démarrage du traitement 5 jours après l’inoculation du virus, alors que les animaux présentent déjà des symptômes. Ce dispositif expérimental ne mimant pas une intervention de PPE, nous n’en commenterons pas les résultats pour le compte de cette revue consacrée à la PPE [24,25].

Il existe malheureusement peu de données cliniques à propos de l’utilisation chez l’humain de mAb114 et REGN-EB3 en PPE. Une série de 23 contacts de cas de MVE en RDC en 2020 a été publiée, dont 4 professionnels de santé et 19 membres de la communauté. Vingt et une personnes ont reçu le mAb114, deux le REGN-EB3. Dix-huit de ces cas d’exposition ont été classés à haut risque. Même si aucune MVE n’a été signalée, cette étude sans groupe témoin ne permet pas d’estimer l’efficacité de l’intervention mise en œuvre [13].

## Discussion - La voie à suivre

Il nous apparaît nécessaire de développer des protocoles de PPE dans la communauté afin de réduire le risque de survenue de la MVE et la mortalité qui lui est associée. Tout bien considéré, les données disponibles sur la PPE sont fragmentaires, particulièrement celles portant sur l’utilisation des anticorps monoclonaux chez l’humain, et elles ne nous permettent pas d’établir avec certitude qu’un outil est plus approprié qu’un autre. Les données précliniques et cliniques disponibles suggèrent cependant que le vaccin rVSVΔG-ZEBOV-GP possède une efficacité limitée en PPE contre la survenue de la MVE, mais une efficacité plus nette pour atténuer la sévérité de la MVE si celle-ci survient.

Pour ce qui concerne les anticorps monoclonaux, les données animales et cliniques suggèrent que leur utilisation confère une protection immédiate et potentiellement plus élevée contre le risque de survenue de la MVE, mais sans qu’on puisse estimer de manière précise et définitive leur efficacité. Une étude clinique observationnelle ou randomisée contrôlée est nécessaire pour permettre de conclure sur ce point.

Les données plaident davantage en faveur du mAb114 et du REGN-EB3 que du rVSVΔG-ZEBOV-GP. Les anticorps monoclonaux sembleraient plus efficaces que le vaccin à prévenir la survenue de la MVE suite à une exposition à EBOV, même s’il n’existe pas de données conclusives. Leur efficacité pourrait être d’autant plus élevée qu’ils sont administrés rapidement après l’exposition. Nous souhaitons donc recommander que la mise à disposition des anticorps monoclonaux soit étendue et qu’elle s’adresse non seulement aux professionnels de santé faisant l’expérience d’un accident d’exposition, mais aussi aux membres de la communauté ayant fait l’objet d’une exposition à haut risque.

Cependant, alors que le vaccin rVSVΔG-ZEBOV peut être facilement et rapidement mis à disposition gratuitement des pays en situation d’épidémie à partir du stock d’urgence géré par l’OMS et ses partenaires de l’International Coordinating Group on Vaccine Provision (ICG), mAb114 et REGN-EB3 sont coûteux, mal distribués et difficiles d’accès. Le prix payé par le gouvernement américain pour doter son stock stratégique national de milliers de doses de REGN-EB3 est estimé à au moins 6 900 US$ par traitement [18,26]. Même si l’OMS dispose également depuis septembre 2025 d’un stock d’environ 500 traitements de REGN-EB3, cette dotation pourrait rapidement s’avérer insuffisante si le traitement venait à être utilisé à des fins de PPE. En outre, les anticorps monoclonaux REGN-EB3 et mAb114 nécessitant une administration par voie intraveineuse, leur utilisation à large échelle à visée prophylactique présenterait des défis en termes de faisabilité opérationnelle. Dans ces conditions, il ne s’agirait pas d’administrer le mAb114 ou le REGN-EB3 en PPE à tous les contacts d’un cas, mais seulement à ceux qui sont le plus à risque de développer la maladie. À cet effet, il est urgent de clarifier et d’optimiser les critères d’appréciation du niveau de risque d’exposition afin d’offrir les anticorps monoclonaux en PPE à ceux qui en ont le plus besoin.

En outre, il reste à déterminer comment associer immunisation active et passive dans le cadre de la PPE contre Ebola. Alors que les anticorps monoclonaux ont précisément pour cible l’antigène vaccinal (EBOV-GP), un effet antagoniste des deux interventions combinées est-il possible, conduisant à une plus faible immunogénicité du vaccin ? Ou bien assisterait-on à un effet synergique, contribuant à renforcer l’effet protecteur ? Plusieurs schémas de combinaison sont envisageables : anticorps en PPE et primo-vaccination en même temps, avec potentiellement une deuxième dose vaccinale plus tard au cas où l’efficacité de la primo-vaccination serait atténuée par les anticorps; anticorps en PPE et primo-vaccination différée d’un jour ou plus, la durée optimale devant être déterminée expérimentalement.

Des recherches sont actuellement en cours pour mieux évaluer l’interaction entre le vaccin et les anticorps monoclonaux et envisager leur utilisation combinée, notamment une étude prospective d’immunogénicité chez des volontaires sains [1]. Alors qu’une publication récente a montré que les anticorps développés naturellement après une infection asymptomatique à EBOV n’inhibent pas l’immunogénicité du vaccin [28], nous espérons que l’utilisation concomitante ou rapprochée des anticorps monoclonaux et du vaccin ne compromettra pas la qualité de la réponse vaccinale. Enfin, l’amélioration des protocoles de PPE n’est pas tout. Elle doit s’accompagner d’une amélioration opérationnelle du suivi des contacts, pour identifier plus rapidement les contacts à haut risque d’exposition et leur permettre un accès rapide aux soins en cas d’apparition de symptômes. De surcroît, alors qu’il existe déjà un stock d’urgence de vaccins contre Ebola, il est essentiel de mettre en place au bénéfice des pays affectés et des organisations internationales un stock similaire en anticorps monoclonaux. Ce stock devra être suffisamment doté pour servir les besoins de la PPE chez les sujets à haut risque, et pas seulement pour le traitement des cas confirmés de MVE.

## Conclusion

Bien qu’il soit important de mener d’autres recherches cliniques pour optimiser les protocoles PPE en milieu communautaire et comparer de manière rigoureuse les performances respectives du rVSVΔG-ZEBOV-GP, du mAb114 et du REGN-EB3, nous pensons que les membres de la communauté exposés à EBOV présentant un risque très élevé de développer la MVE devraient se voir offrir la possibilité d’une PPE avec des anticorps monoclonaux, comme c’est déjà le cas pour les travailleurs du secteur de la santé ou les nouveau-nés dont la mère a été infectée par EBOV.

## Financement

Cette étude n’a bénéficié d’aucun financement.

## Contributions des auteurs et autrices

Julien POTET, Ismael ADJAHO, Kerry DIERBERG, Armand SPRECHER, Rebecca Marie COULBORN : conception de l’étude, prospection bibliographique, définition de la méthodologie, analyse des données, validation des données, interprétation des résultats, rédaction du manuscrit, relecture et validation du manuscrit.

## Déclaration de liens d’intérêts

Aucun lien d’intérêt n’a été déclaré.
