# Supplementary material for: Post-exposure prophylaxis against Ebola virus: arguments for expanding the role of monoclonal antibodies in a context of limited access
Source: Med Trop Sante Int. 2026 Mar 31;6(1):mtsi.v6i1.2026.743. [Article in French] doi: 10.48327/mtsi.v6i1.2026.835 (PMC13216899; doi:10.48327/mtsi.v6i1.2026.835)
Supplement: Related texts

# Research article: Post-exposure prophylaxis against Ebola virus: arguments for expanding the role of monoclonal antibodies in a context of limited access

## Introduction and methods

The search for new tools to combat Ebola virus disease (EVD) has made considerable progress over the past decade. Clinical trials of several vaccine and therapeutic candidates were conducted during Ebola (EBOV) epidemics in West Africa between 2013 and 2016, and in the Democratic Republic of Congo (DRC) between 2018 and 2020. Today, there are four products recommended by the World Health Organization (WHO): two vaccines and two monoclonal antibody treatments [21,22]. Of the two approved vaccines, the one that has been prioritised in terms of epidemic response is rVSVΔG-ZEBOV-GP, which is suitable for this type of context due to its single-dose formulation capable of inducing rapid protection [23].

In infectious disease medicine, therapeutic antibodies are frequently used for prophylactic purposes, either in combination with vaccines or on their own [9]. However, at present, the prophylactic use of monoclonal antibodies against Ebola is very limited: protocols and recommendations exist, but only for healthcare professionals who have been exposed to EBOV [20] and for newborns less than 7 days old born to mothers infected with EBOV [22]. Their use among members of the community following high-risk exposure to EBOV is not yet recommended. According to the most recent definitions, in addition to newborns born to infected mothers, there are three categories of individuals considered to be at high risk: 1) children breastfed by a mother infected with EBOV; 2) individuals in direct contact with a person with confirmed EBOV infection presenting with “wet” symptoms such as diarrhoea, vomiting or external haemorrhage, or in contact with their bodily fluids; 3) individuals in direct contact with the corpse of a person with confirmed or probable EBOV infection [12]. In this article, in addition to a recently published literature review [12], we propose to analyse the available data on rVSVΔG-ZEBOV-GP and the monoclonal antibodies mAb114 and REGN-3, with a view to their potential use in post-exposure prophylaxis (PEP). The main characteristics of these three products are presented in Table I. Our narrative review, based on peer-reviewed articles and guidelines proposed by organisations responding to Ebola epidemics, aims to answer the following question: which tools are likely to be most effective for use in PEP, the rVSVΔG-ZEBOV-GP vaccine, the monoclonal antibodies mab114 and REGN-EB3, or a combination of antibodies and vaccine? Here we detail the available data and outstanding questions, while acknowledging that our literature search is not necessarily exhaustive and that other drugs, including new antivirals such as obeldesivir, are also being proposed as candidates for PEP. Since monoclonal antibodies act as passive immunisation, we argue that they should be offered after high-risk exposure to EBOV, not only to healthcare workers but also to members of the community.

**Table I:** Main characteristics of products considered for EVD PEP

	Availability and costs	Storage conditions	Shelf life	Method of administration
**REGN-EB3**	Emergency stockpile in the United States. Price paid by the US government estimated at least US$6,900 per treatment. Emergency stockpile of 500 treatments donated by Regeneron to the WHO in September 2025	Store between 2°C and 8°C	36 months	Intravenous
**mAb114**	No emergency stockpile at the WHO mAb114 donation programme developed by Ridgeback-Bio, but origin of doses and quantities available unknown	Store between 2°C and 8°C	Varies depending on the batch used	Intravenous
**rVSVΔG- ZEBOV-GP**	Emergency stockpile of 500,000 doses at the WHO (ICG), funded by GAVI. Price paid by GAVI: US$99 per dose	Store between -80°C and -60°C, with a tolerance of 14 days between 2°C and 8°C	36 months	Intramuscular

## Results

### rVSVΔG-ZEBOV-GP in PEP

The rVSVΔG-ZEBOV-GP vaccine, marketed by Merck Sharp & Dohme (MSD) under the brand name Ervebo, uses vesicular stomatitis virus (VSV) as a replicating vector and carrier for the antigen, EBOV glycoprotein (EBOV-GP). It has been demonstrated that the vaccine confers a very high level of protection against the onset of EVD 10 days after administration [10,16]. The vaccine is mainly used during epidemics as part of a “ring” vaccination strategy around a case, among the case’s contacts and the contacts of those contacts. In fact, among the contacts receiving the vaccine in this way, there are people who have recently been exposed to EBOV, some of whom may have a high risk of developing disease. These may include family members of the patient who cared for them without any protective equipment before the diagnosis was made, or participants in unsafe funeral rites during which the body of the deceased was touched.

There are reasons for concern that rVSVΔG-ZE-BOV-GP may have limited efficacy in PEP among contacts who have been recently exposed to EBOV, given that vaccine antibodies are generally detected between 7 and 14 days after vaccination in most subjects [11], and the median incubation period for the disease is estimated by some authors to be 10 days [14]. However, it has been shown that the rVSV viral vector stimulates innate immunity, which could rapidly confer some protection before the antigen-specific immune response can take effect [17]. This is suggested by animal data: when the vaccine is administered to macaques less than 24 hours after they have been inoculated with a dose of 1,000 pfu of EBOV, which is lethal in the absence of medical intervention, between one-third and two-thirds of the monkeys survive, depending on the experiment [7,15]. The protection is significant, but limited.

On the clinical side, eight cases of healthcare professionals who received the rVSVΔG-ZEBOV-GP vaccine following accidental exposure to EBOV at varying levels of risk were reported in 2017. No infections were reported [8]. More recently, among 45 people in the United Kingdom who had been in contact with a previously recovered patient suffering from a relapse of EVD, 26 received the vaccine. No infections were detected in either the vaccine group or the control group. As the characteristics of the index patient (a rare case of relapse) were very specific and the risk of exposure was considered moderate, these data should be interpreted with great caution [6]. A retrospective case-control study with negative tests, conducted in the DRC between 2018 and 2019 [16], provides some clarity. This study focuses on contacts who developed symptoms suggestive of EVD and were therefore admitted to a specialised Ebola treatment centre for diagnostic evaluation and care: the “control” group consists of contacts who ultimately tested negative for EBOV, while the “case” group consists of contacts who actually developed EVD. Unsurprisingly, the proportion of people who received the vaccine more than 10 days before the onset of symptoms in the control group (ultimately negative for EBOV) was significantly higher than in the case group (ultimately positive for EBOV). The authors therefore estimate that the effectiveness of rVSVΔG-ZEBOV-GP against EVD 10 days or more after vaccination is 84% (95% CI [70,92]). However, the difference between the control and case groups was much smaller in individuals vaccinated between 3 and 9 days before the onset of symptoms. The protection conferred by rVSVΔG-ZEBOV-GP against the onset of EVD within 3-9 days after vaccination is therefore estimated to be only 16% (95% CI [35,48]). Considering that the average incubation period for EVD is approximately 10 days [14], it can be assumed that a large number of these cases had been vaccinated after being exposed to EBOV. The small sample size and wide confidence interval call for caution, as does the likely difference in vaccine efficacy between individuals vaccinated 3 days before the onset of symptom and those vaccinated 9 days before. Nevertheless, these data, taken as a whole, indirectly suggest that the vaccine would have limited efficacy against the development of EVD if used in the context of PEP.

However, another retrospective study in the DRC, this time only among patients with EVD, suggests that recent vaccination, between 3 and 9 days before the onset of symptoms, is associated with a risk of death that is less than half the risk of death observed in unvaccinated patients with EVD (adjusted relative risk: 0.44 [0.29–0.65, p=0.0001]) [4].

### REGN-EB3 and mAb114 in PEP

Various passive immunisation approaches have been proposed for the treatment or prevention of EVD, including the use of convalescent patient sera or animal sera [2,27]. However, the two approved treatments for EVD consist of monoclonal antibodies targeting the EBOV glycoprotein. These are mAb114 (ansuvimab, brand name Ebanga, marketed by Ridgeback-Biotherapeutics) and REGN-EB3 (atoltivimab + maftivimab + odesivimab, brand name Inmazeb, marketed by Regeneron), both of which have demonstrated efficacy as treatment for EVD. They must be administered intravenously.

It has been shown in both humans and animals that the later treatment targeting EBOV and other filoviruses is started after the onset of symptoms, the less effective they are [5,19]. There therefore appears to be a time-dependent gradient of efficacy. Following this line of reasoning, it can be hypothesised that the efficacy of mAb114 and REGN-EB3 in preventing EVD or mitigating its severity if the disease does occur should be very high if they are administered as PEP.

The results obtained in animals support this hypothesis. The administration of three consecutive doses of mAb114 to rhesus macaques 24 hours, 48 hours and then 72 hours after they had been inoculated with a dose of 1,000 pfu of EBOV resulted in a 100% survival rate, with no clinical signs, but without preventing the presence of very low viraemia [3]. To our knowledge, these are the only data available in non-human primates with an experimental design mimicking PEP, in which treatment begins very early after virus inoculation and before the onset of symptoms. Other experiments with REGN-EB3 and mAb114 have been published in non-human primates, with treatment starting 5 days after virus inoculation, when the animals are already showing symptoms. As this experimental design does not mimic a PEP intervention, we will not comment on the results in this review [24,25].

Unfortunately, there is little clinical data on the use of mAb114 and REGN-EB3 in humans for PEP. A series of 23 contacts of EVD cases in the DRC in 2020 has been published, including 4 healthcare workers and 19 community members. Twenty-one people received mAb114 and two received REGN-EB3. Eighteen of these exposure cases were classified as high risk. Although no EVD cases were reported, this uncontrolled study does not allow for an estimation of the effectiveness of the intervention implemented [13].

## Discussion - The way forward

We believe it is necessary to develop PEP protocols in the community in order to reduce the risk of EVD and associated mortality. All things considered, the available data on PEP are fragmentary, particularly those relating to the use of monoclonal antibodies in humans, and do not allow us to establish with certainty that one tool is more appropriate than another. However, the available preclinical and clinical data suggest that the rVSVΔG-ZEBOV-GP vaccine has limited efficacy as PEP against the occurrence of EVD but is more effective in mitigating the severity of EVD if it does occur.

With regard to monoclonal antibodies, animal and clinical data suggest that their use confers immediate and potentially higher protection against the risk of EVD, but their efficacy cannot be accurately and definitively estimated. An observational or randomised controlled clinical trial is needed to reach a conclusion on this point. The data are more favourable for mAb114 and REGN-EB3 than for rVSVΔG-ZEBOV-GP. Monoclonal antibodies appear to be more effective than the vaccine in preventing the onset of EVD following exposure to EBOV, although there are no conclusive data. Their efficacy may be higher when administered rapidly after exposure. We therefore recommend that the availability of monoclonal antibodies be expanded to include not only healthcare professionals who have been exposed, but also members of the community who have been at high risk of exposure.

However, while the rVSVΔG-ZEBOV vaccine can be easily and quickly made available free of charge to countries experiencing an epidemic from the emergency stockpile managed by the WHO and its partners in the International Coordinating Group on Vaccine Provision (ICG), mAb114 and REGN-EB3 are expensive, poorly distributed and difficult to access. The price paid by the US government to stock its national strategic reserve with thousands of doses of REGN-EB3 is estimated at least US$6,900 per treatment [18,26]. Although the WHO has also had a stockpile of approximately 500 treatments of REGN-EB3 since September 2025, this supply could quickly prove insufficient if the treatment were to be used for PEP purposes. Furthermore, as the monoclonal antibodies REGN-EB3 and mAb114 require intravenous administration, their widespread use for prophylactic purposes would present challenges in terms of operational feasibility. Under these conditions, mAb114 or REGN-EB3 would not be administered as PEP to all contacts of a case, but only to those most at risk of developing the disease. To this end, there is an urgent need to clarify and optimise the criteria for assessing the level of exposure risk in order to offer monoclonal antibodies as PEP to those who need them most. In addition, it remains to be determined how to combine active and passive immunisation in the context of Ebola PEP. Given that monoclonal antibodies specifically target the vaccine antigen (EBOV-GP), is it possible that the two interventions combined could have an antagonistic effect, leading to lower vaccine immunogenicity? Or would there be a synergistic effect, helping to strengthen the protective effect?

Several combination regimens are possible: PEP antibodies and primary vaccination at the same time, with potentially a second vaccine dose later if the efficacy of the primary vaccination is attenuated by the antibodies; PEP antibodies and primary vaccination delayed by one day or more, with the optimal duration to be determined experimentally.

Research is currently underway to better assess the interaction between the vaccine and monoclonal antibodies and to consider their combined use, including a prospective immunogenicity study in healthy volunteers [1]. While a recent publication has shown that antibodies developed naturally after asymptomatic EBOV infection do not inhibit vaccine immunogenicity [28], we hope that the concomitant or close use of monoclonal antibodies and the vaccine will not compromise the quality of the vaccine response.

Finally, improving PEP protocols is not enough. It must be accompanied by operational improvements in contact tracing, to identify high-risk contacts more quickly and enable them to access care rapidly if symptoms appear. Furthermore, while there is already an emergency stockpile of Ebola vaccines, it is essential to establish a similar stockpile of monoclonal antibodies for the benefit of affected countries and international organisations. This stockpile must be large enough to meet the PEP needs of individuals at high-risk for developing disease, and not just for the treatment of confirmed cases of EVD.

## Conclusion

Although further clinical research is needed to optimise PEP protocols in community settings and rigorously compare the respective performances of rVSVΔG-ZEBOV-GP, mAb114 and REGN-EB3, we believe that community members exposed to EBOV who are at very high risk of developing EVD should be offered the option of PEP with monoclonal antibodies, as is already the case for healthcare workers or newborns whose mothers have been infected with EBOV.

## Funding

This study received no funding.

## Authors’s contributions

Julien POTET, Ismael ADJAHO, Kerry DIERBERG, Armand SPRECHER, Rebecca Marie COULBORN: study design, literature review, methodology development, data analysis, data validation, interpretation of results, manuscript drafting, manuscript review and approval.

## Conflicts of interest

The authors declare no conflicts of interest.
